# Recovery and Growth Potential of *Listeria monocytogenes* in Temperature Abused Milkshakes Prepared from Naturally Contaminated Ice Cream Linked to a Listeriosis Outbreak

**DOI:** 10.3389/fmicb.2016.00764

**Published:** 2016-05-18

**Authors:** Yi Chen, Emma Allard, Anna Wooten, Minji Hur, Ishani Sheth, Anna Laasri, Thomas S. Hammack, Dumitru Macarisin

**Affiliations:** ^1^Office of Regulatory Science, Center for Food Safety and Applied Nutrition, Food and Drug Administration, College ParkMD, USA; ^2^College of Life Sciences and Agriculture, University of New Hampshire, DurhamNH, USA; ^3^Joint Institute for Food Safety and Applied Nutrition, University of Maryland, College ParkMD, USA; ^4^Department of Food Science, Gachon UniversitySeoul, South Korea

**Keywords:** *L. monocytogenes*, ice cream, milkshake, growth potential, temperature abuse, outbreak

## Abstract

The recovery and growth potential of *Listeria monocytogenes* was evaluated in three flavors of milkshakes (vanilla, strawberry, and chocolate) that were prepared from naturally contaminated ice cream linked to a listeriosis outbreak in the U.S. in 2015, and were subsequently held at room temperature for 14 h. The average lag phase duration of *L. monocytogenes* was 9.05 h; the average generation time was 1.67 h; and the average population level increase per sample at 14 h was 1.14 log CFU/g. Milkshake flavors did not significantly affect these parameters. The average lag phase duration of *L. monocytogenes* in milkshakes with initial contamination levels ≤ 3 CFU/g (9.50 h) was significantly longer (*P* < 0.01) than that with initial contamination levels > 3 CFU/g (8.60 h). The results highlight the value of using samples that are contaminated with very low levels of *L. monocytogenes* for recovery and growth evaluations. The behavior of *L. monocytogenes* populations in milkshakes prepared from naturally contaminated ice cream linked to the listeriosis outbreak should be taken into account when performing risk based analysis using this outbreak as a case study.

## Introduction

In March 2015, a listeriosis outbreak (outbreak I) was reported from a hospital (hospital X) involving five highly susceptible elderly patients who were hospitalized for other medical conditions prior to exposure to *Listeria monocytogenes* (Centers for Disease Control and Prevention, 2015). DNA fingerprinting [pulsed field gel electrophoresis (PFGE) and whole genome sequencing (WGS)] of *L. monocytogenes* isolates from these patients and various ice cream products linked four patients to the consumption of three flavors of milkshakes (vanilla, chocolate, and strawberry) served at lunch or dinner in hospital X and prepared with the contaminated ice cream scoops that were produced in production line A (Centers for Disease Control and Prevention, 2015; Karl Klontz, personal communication). It is expected that very little post-contamination growth occurred in ice cream because this product is kept frozen all along the production and distribution chain. Thus, enumeration of *L. monocytogenes* in these products would provide a relatively clear estimation of the amount of *L. monocytogenes* consumed by the case patients and shed some light on the risk associated with *L. monocytogenes* contamination and its infective dose. Therefore, Chen (2015) enumerated the levels of *L. monocytogenes* in 2,320 individually wrapped scoops of ice cream produced in production line A. The enumeration study demonstrated that *L. monocytogenes* was present in 99.4% of all tested products produced between November 2014 and March 2015, and among them *L. monocytogenes* was present in 100% of tested products produced between November 2014 and January 2015. The levels of *L. monocytogenes* in individual scoops were below 20 and 50 most probable number (MPN)/g in 92.3 and 98.4% of tested scoops, respectively; and they were homogeneously low among different production dates with a geometric mean concentration of 0.15 to 7.1 MPN/g (Chen, 2015).

However, the case patients consumed milkshakes prepared from contaminated ice cream scoops, and thus, the enumeration data cannot be directly used for risk assessment without taking into account the possible growth of *L. monocytogenes* in milkshakes prior to human consumption. Another listeriosis outbreak (outbreak II) linked to contaminated ice cream products occurred in another state in 2015 (Rietberg et al., 2015), involving two patients from a hospital (hospital Y) who were served milkshakes prepared from contaminated ice cream. No evidence of temperature abuse of milkshakes in hospital X or Y was reported (Centers for Disease Control and Prevention, 2015; Rietberg et al., 2015), and no samples were available from outbreak II (Rietberg et al., 2015). Milkshake is a commonly consumed commodity, and these two outbreaks called for studies on the behavior of *L. monocytogenes* in milkshakes prepared from ice cream products, especially those naturally contaminated with *L. monocytogenes*. However, such studies have never been reported. We were able to obtain ice cream samples produced from the production line that was implicated in outbreak I, and therefore, the objective of the present study was to evaluate the recovery and growth potential of *L. monocytogenes* in milkshakes prepared from naturally contaminated ice cream scoops linked to outbreak I. Assuming an extreme scenario that a milkshake was unintentionally left at room temperature for an extended period of time, we evaluated the recovery and growth of *L. monocytogenes* in the course of 14 h at room temperature.

## Materials and Methods

### Ice Cream Samples

Individually wrapped scoops of ice cream produced from production line A were made available by the company. The scoops produced between November 2014 and January 2015 were used to prepare the milkshakes.

### Preparation of Milkshakes

Vanilla, strawberry and chocolate milkshakes were prepared using the recipe from hospital X. Vanilla milkshakes were made from two scoops of ice cream (80–85 g/scoop) and 118 mL of 1% fat milk. Strawberry milkshakes were made from two scoops of ice cream, 118 mL of 1% fat milk and 15 mL of strawberry syrup. Chocolate milkshakes were made from two scoops of ice cream, 118 mL of 1% fat milk and 15 mL of chocolate syrup. Milk, strawberry syrup, and chocolate syrup were purchased from a local supermarket. Ice cream scoops were briefly left at room temperature to soften, and milkshakes were then prepared using a sterilized commercial drink mixer (Model, HMD200, Hamilton Beach Inc., Southern Pines, NC, USA). For each milkshake flavor, 10 milkshake samples were prepared from randomly picked ice cream scoops, and held at room temperature (22.5 ± 0.5°C). To facilitate thorough mixing of the milkshakes, the entire portion of milkshakes were aseptically transferred to sterilized stainless steel laboratory blenders (Model, Waring^®^ 7011S, Conair Corporation, East Windsor, NJ, USA) after preparation, and blended prior to each hourly sampling. The temperature increases of 10 randomly selected milkshakes used for growth curve construction were measured every half hour using thermometers certified by U.S. National Institute of Standards and Technology (Cat. #ACC10033BLSFC, Thermo Fisher Scientific Inc., Waltham, MA, USA). Ten additional vanilla milkshakes were prepared as described above, aseptically transferred to plastic cups and incubated at room temperature. Their temperatures were monitored to investigate the effect of container material and the absence of blending on the temperature increase. These 10 additional samples were not used to evaluate the growth potential of *L. monocytogenes* and thus were not subject to hourly blending.

### Growth Curves

Enumeration of *L. monocytogenes* in milkshakes was conducted hourly in the course of 14 h. During the first 10 h of sampling 2 g of each milkshake was directly plated onto 5 RAPID’*L. mono* agar (Cat. No. 3563694, Bio-Rad Laboratories, Hercules, CA, USA) plates (400 μl/plate) using the easySpiral^®^ automatic spiral plater (Interscience, Inc., France) set to constant volume plating, and this plating yielded a limit of detection (LOD) of 0.5 CFU/g. After 10 h, 1 g of samples was directly plated onto 5 RAPID’*L. mono* agar plates (200 μl/plate), and this plating yielded a LOD of 1 CFU/g. All plates were left to dry before incubation. Representative colonies were confirmed according to the *L. monocytogenes* chapter of the FDA *Bacteriological Analytical Manual* (Hitchins et al., 2016). The *L. monocytogenes* level change (in CFU/g) of each sample was then used to construct the growth curve. The lag phase was determined as the time for the initial population level to increase twofold (Buchanan and Solberg, 1972). The data were not transferred to log values due to very low values (<10 CFU/g) of most of the data points. The generation time was calculated as the time required for cells to double during the exponential phase.

### Statistical Analysis

Comparison of lag phase durations and generation times of *L. monocytogenes* in different flavors of milkshakes with different initial levels of *L. monocytogenes* were performed using one way ANOVA analysis (Bewick et al., 2004) or *t*-test (Whitley and Ball, 2002).

## Results and Discussion

**FIGURE 1:**
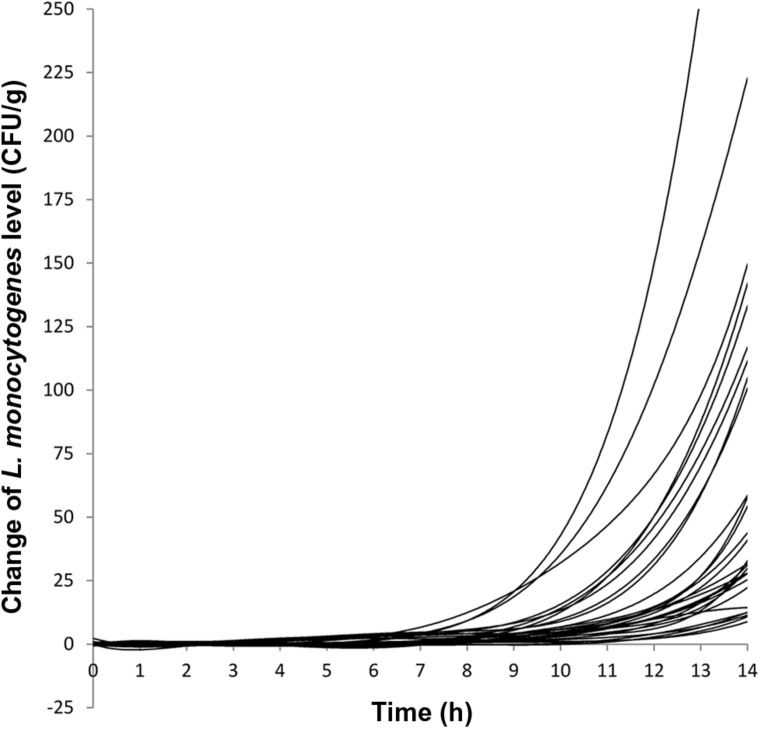
**Growth curves of 30 milkshake samples (10 vanilla milkshakes, 10 strawberry milkshakes, and 10 chocolate milkshakes) held at 22.5°C for 14 h.** Each line represents the growth curve of a milkshake sample.

The growth evaluation was performed on naturally contaminated ice cream products that had varying initial levels of *L. monocytogenes*. Combining results from milkshakes of all three flavors, the initial *L. monocytogenes* levels ranged from 1 to 20.5 CFU/g with 50% (15/30) of the milkshakes having initial *L. monocytogenes* levels ≤ 3 CFU/g and 76.7% (23/30) of the milkshakes having initial *L. monocytogenes* levels ≤ 5 CFU/g. The final *L. monocytogenes* population after 14 h of incubation at 22.5°C ranged from 9 to 422 CFU/g with 73.3% (22/30) of the milkshakes having final *L. monocytogenes* levels ≤ 100 CFU/g and 93.3% (28/30) of the milkshakes having final *L. monocytogenes* levels ≤ 150 CFU/g. Two samples having initial *L. monocytogenes* levels of 12 and 20.5 CFU/g yielded final levels of 233 and 422 CFU/g, respectively. The average level increase per sample at 14 h was 1.23 ± 0.26 (average ± standard deviation), 1.01 ± 0.19, and 1.19 ± 0.15 log CFU/g for vanilla, strawberry, and chocolate milkshakes, respectively, with an average of 1.14 ± 0.22 log CFU/g for all milkshakes (**Table 1**). This indicated that the milkshakes were in the early stage of the exponential phase, and therefore, the growth curve was presented as the change of CFU/g and lag phase duration was determined as the time required for initial cell level to increase twofold (**Figure 1**; Buchanan and Solberg, 1972). The average lag phase durations of *L. monocytogenes* were 8.85 ± 0.78, 9.50 ± 0.82, and 8.80 ± 0.95 h for vanilla, strawberry, and chocolate milkshakes, respectively (**Table 1**; **Figure 1**), which were not statistically different (*P* > 0.05) from each other. The average lag phase duration was 9.05 ± 0.88 h for all milkshakes (**Table 1**). The average generation times of *L. monocytogenes* were 1.65 ± 0.39, 1.79 ± 0.36, and 1.57 ± 0.18 h for vanilla, strawberry, and chocolate milkshakes, respectively (**Table 1**), which were not statistically different (*P* > 0.05) from each other. The average generation time was 1.67 ± 0.33 h for all milkshakes. Because of the relatively long lag phase, no growth of *L. monocytogenes* was observed for 7.30 ± 0.79, 8.05 ± 0.96, and 7.40 ± 0.70 h for vanilla, strawberry, and chocolate milkshakes, respectively (**Table 1**; **Figure 1**). To our knowledge, this is the first report of the recovery and growth potential of *L. monocytogenes* in milkshakes prepared with ice cream products that were naturally contaminated with very low levels of bacteria and produced from a production line that was implicated in a listeriosis outbreak. The lag phase duration and generation time of *L. monocytogenes* determined in the present study contributed to a better understanding of the behavior of *L. monocytogenes* in the temperature abused milkshake prepared with naturally contaminated ice cream. The data can be combined with previously generated enumeration data for risk based characterization of *L. monocytogenes* contamination in this commodity. There is a possibility that the average generation time of the entire exponential phase may be different from the generation time observed in the present study; however, we chose to calculate the lag phase duration and generation time within 14 h of incubation because that value was relevant in assessing the recovery and growth of *L. monocytogenes*, had the milkshakes been unintentionally left at room temperature during the day of serving. A probabilistic analysis between direct plating and MPN for the ice cream samples showed agreement of the two methods in 96.1% of the samples with direct plating providing an underestimate in 0.8% of the samples and MPN providing an underestimate in 3.1% of the samples. This indicated that the injury status of *L. monocytogenes* in these ice cream products did not prevent the cells from recovering and growing on RAPID’*L. mono* agar (Chen, 2015). WGS analysis using the Center for Food Safety and Applied Nutrition (CFSAN) Single Nucleotide Polymorphism (SNP) pipeline (Pettengill et al., 2014; Davis et al., 2015) showed that the randomly picked isolates from these ice cream products all matched the clinical specimen of the patients in hospital X (unpublished data).

**Table 1:** Lag phase duration, generation time, time before growth was observed, level increase per sample for the 30 milkshakes (10 vanilla milkshakes, 10 strawberry milkshakes, and 10 chocolate milkshakes) analyzed in the present study.

Milkshake flavor	Lag phase duration (h)	Generation time (h)	Time before growth was observed (h)	Level increase per sample at 14 h (Log CFU/g)
Vanilla	8.85 ± 0.78	1.65 ± 0.39	7.30 ± 0.79	1.23 ± 0.26
Strawberry	9.50 ± 0.82	1.79 ± 0.36	8.05 ± 0.96	1.01 ± 0.19
Chocolate	8.80 ± 0.95	1.57 ± 0.18	7.40 ± 0.70	1.19 ± 0.15
Total	9.05 ± 0.88	1.67 ± 0.33	7.58 ± 0.86	1.14 ± 0.22

**FIGURE 2:**
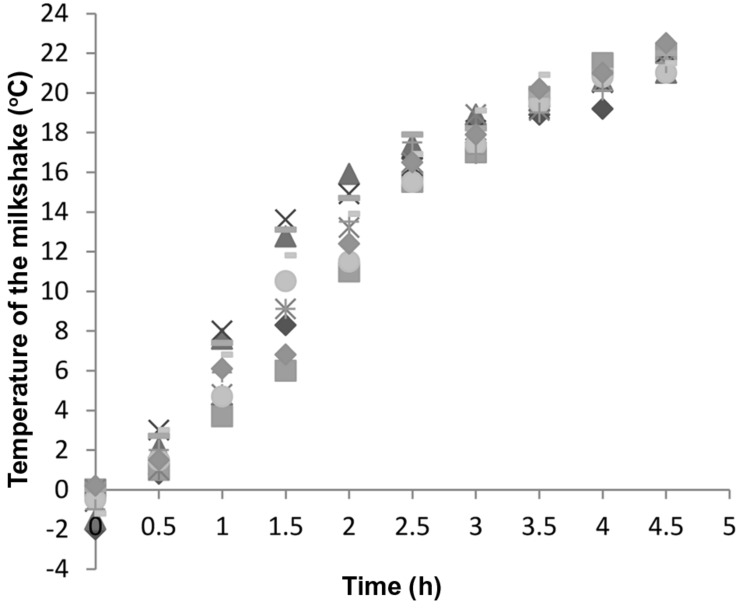
**Temperature increase of 10 randomly selected milkshakes held in stainless steel containers at 22.5°C that were used for growth curve construction.** Different symbols represent different milkshake samples.

It is very important to keep in mind that during the first few hours of the exposure to room temperature, the temperature of the milkshakes was low. Milkshakes were –2 to 0°C when freshly prepared and reached 17 to 19°C after being held at room temperature for 3 h (**Figure 2**) in stainless steel cups, and thus, after milkshakes reached room temperature, it took *L. monocytogenes* another 6.05 h to reach the exponential phase. Milkshakes in plastic cups that were not subject to hourly blending reached 17 to 19°C after 4 h at room temperature. Therefore the use of stainless steel container and hourly blending facilitated a slightly faster temperature increase of milkshakes than plastic cups without hourly blending.

Studies which examined growth kinetics of *L. monocytogenes* in various enrichment broths and foods under various conditions often had the technical limitation (Gnanou Besse et al., 2006) set by the sensitivity of enumeration methods. In some cases, the variability in the enumeration of low levels of bacterial cells could compromise the reliability of any statistical analysis (Duffy et al., 1994). This is probably why quite a few studies used foods artificially inoculated with 10^2^ to 10^4^ CFU/g or even higher levels of bacteria for the convenience of subsequent enumeration and statistical analysis (Xanthiakos et al., 2006; Panagou and Nychas, 2008; Schvartzman et al., 2014; Huang et al., 2015; Luo et al., 2015). However, these inoculum sizes do not reflect the low bacterial contamination levels usually found in food. Indeed, if the bacterial levels are low, a large number of agar plates or an MPN scheme with a large number of MPN tubes per level and a large number of biological replicates would have to be used to accurately determine the lag phase duration. This would be impractical for hourly monitoring of bacterial growth, especially for an extended period of time. The milkshake samples in the present study could be homogenized with no dilutions in any buffer; and they were viscous and could stay off the edge of the plates. As a result, as much as 400 μl of milkshake could be evenly spread onto one plate with the help of the automatic spiral plater, and this allowed us to plate up to 2 g per sample and to obtain reliable data to perform statistical analysis.

The average lag phase duration of *L. monocytogenes* in 15 milkshakes having initial *L. monocytogenes* levels ≤ 3 CFU/g was 9.50 ± 0.76 h, and it was significantly higher (*P* < 0.01) than that of 15 milkshakes having initial *L. monocytogenes* levels > 3 CFU/g, which was 8.60 ± 0.78 h. Among the 15 milkshakes having initial *L. monocytogenes* levels > 3 CFU/g, eight milkshakes had initial *L. monocytogenes* levels of 3.5 to 5 CFU/g and seven milkshakes had initial *L. monocytogenes* levels of 5.5 to 20.5 CFU/g. Their average lag phase durations were 8.44 ± 0.78 and 8.79 ± 0.81 h, respectively, which were not significantly different (*P* > 0.05) from each other. The enumeration study revealed that 7.7, 1.6, and 0.2% of all tested ice cream scoops had *L. monocytogenes* of more than 20, 50, and 100 MPN/g, respectively (Chen, 2015). The milkshakes were made of two 80–85 g scoops and then diluted in 118 mL of milk, and thus, in order to obtain milkshakes containing *L. monocytogenes* of more than 30 CFU/g the addition of numeric values of the *L. monocytogenes* levels in the two scoops needed to exceed 100. The probability of this happening was very low, and therefore, we would not have been able to obtain sufficient biological replicates of milkshakes containing *L. monocytogenes* of more than 30 CFU/g even if we had prepared a much larger number of milkshakes. Robinson et al. (2001) showed that when the inoculum levels of *L. monocytogenes* were below 100 to 1,000 cells per sample, the lag time increased as the inoculum size decreased, especially under suboptimal growth conditions, partially due to the variability in the lag phase of individual cells; and the effect of inoculum size on lag phase duration started to disappear when the inoculum levels were higher. Aguirre et al. (2013) found that under experimental conditions, the lag phase durations of *L. innocua* with initial levels of 0.7 to 20 CFU/sample were higher than those with initial levels of 20 to 200 CFU/sample, and there was no statistical difference in lag phase durations among populations with initial levels between 20 and 200 CFU/sample. This phenomenon was also observed by Baranyi (1998) and Pin and Baranyi (2006) in other bacteria. The results from different studies may not be directly compared due to differences in the *Listeria* species/strains, growth media, and applied methodologies, however, they could partially explain why we observed a longer lag phase duration (*P* < 0.01) of cells in milkshakes with initial levels ≤ 3 CFU/g than that of cells with initial levels between 3 and 20.5 CFU/g, and no significant difference (*P* > 0.05) in lag phase durations between cells with initial levels of 3.5 to 5 CFU/g and those with initial levels of 5.5 to 20.5 CFU/g. The finding in the present study, combined with those reported previously, suggests that under certain growth conditions the average lag phage duration of cells that are sparsely distributed in foods could be significantly longer than that indicated from foods inoculated with high levels of inoculum.

No studies have been performed on the behavior of *L. monocytogenes* in milkshakes made from ice cream, but a few studies analyzed artificially inoculated dairy products. Alavi et al. (1999) reported that the generation time of *L. monocytogenes* with an initial inoculum level of 1,000 CFU/mL in liquid whole milk stored at 22.5°C was 1.40 h. Xanthiakos et al. (2006) reported that with an initial inoculum level of 10^3^ to 10^4^ CFU/mL in pasteurized milk stored at 16°C, the lag phase duration of *L. monocytogenes* was 7.12 h and the generation time was 1.27 h. Panagou and Nychas (2008) found that with an initial inoculum level of 100 CFU/g, *L. monocytogenes* in a vanilla cream dessert-type product held at 15°C had an average lag phase duration of 7.85 h and an average generation time of 1.45 h. Gougouli et al. (2008) studied the behavior of *L. monocytogenes* in artificially inoculated ice cream extensively under multiple chilling and freezing conditions. A pro-longed (500 h) chilling-freezing experiment monitoring the recovery and growth of *L. monocytogenes* every 20 to 40 h demonstrated that *L. monocytogenes* cells in ice cream did not suffer significant injury in freezing conditions up to –30°C, and during consecutive freezing and thawing cycles. This suggested that the relatively long lag phase and generation time determined in the present study were not direct results of cell injury. Due to the overall low prevalence of *L. monocytogenes* in foods, most studies that used naturally contaminated samples could only yield a small number of positive samples and thus not enough biological replicates could be generated (Jorgensen and Huss, 1998; Encinas et al., 1999; Lappi et al., 2004; Beaufort et al., 2007). The 100% prevalence rate of *L. monocytogenes* (Chen, 2015) in the ice cream samples used in the present study allowed the use of a large number of naturally contaminated biological replicates to generate the unique and highly confident research data.

## Conclusion

This is the first report on the characterization of the behavior of *L. monocytogenes* in milkshakes prepared from the ice cream products naturally contaminated with low levels of *L. monocytogenes*. This provides relevant information for future risk assessment using the 2015 U.S. ice cream listeriosis outbreak as a case study, especially since the ice cream scoops used in the present study were produced from the production line that was implicated in the outbreak. The results demonstrated the value of using samples contaminated with very low levels of *L. monocytogenes* to perform recovery and growth evaluations of *L. monocytogenes* in foods. It is important to keep in mind that the rather long lag phase and generation time observed only pertained to the strains/samples in the present study, and our conclusions by no means undermine the critical importance of stringent temperature control by food handlers.

## Author Contributions

YC is the principle investigator (PI) of the project and wrote the manuscript. TH and DM are co-PIs of the project. EA, AW, MH, IS, and AL are research scientists working on the experiments.

## Conflict of Interest Statement

The authors declare that the research was conducted in the absence of any commercial or financial relationships that could be construed as a potential conflict of interest. The findings and conclusions in this report are those of the authors and do not necessarily represent the official position of the U.S. FDA.
